# The Significance of Leucorrhea*From a paper presented by invitation at the Richmond meeting of the Tri-State Medical Association of the Carolinas and Virginia, February 15 to 17.

**Published:** 1910-04

**Authors:** Augustin H. Goelet

**Affiliations:** New York


For Texas Medical Journal.

### The Significance of Leucorrhea.*

BY AUGUSTIN H. GOELET, M. D., NEW YORK.

The neglect of leucorrhea is a grave and unfortunately a common error that is unpardonable because it involves the health, happiness and often the life of the patient. The character and source of a vaginal discharge should always be investigated by the physician who encounters it and the patient should never be dis-

*From a paper presented by invitation at the Richmond meeting of the Tri-State Medical Association of the Carolinas and Virginia, February 15 to 17.

missed with a prescription for a vaginal wash to be used unintelligently and indifferently because the true significance of the discharge is not appreciated.

Education of patients to an appreciation of the possible significance of leucorrhea in *childhood before puberty, in young women before marriage, in married women, and in women past the menopause* is particularly insisted upon.

The danger to the woman in after life of disregarding or neglecting a vulvo-vaginitis of childhood that may be derived from infection of a serious character is forcibly emphasized, as is also the necessity of investigating the leucorrhea of young girls after puberty and before marriage. These latter are the cases most often neglected because of false modesty on the part of both the mother and daughter, shared only too often by the physician. It too often happens that the unsuspecting mother and physician permit neglect of a vulvo-vaginitis of gonorrheal origin because a proper examination is not insisted upon.

The leucorrhea of recently married women, as is pointed out, is often the result of infection from the husband who has a gleet or chronic prostatitis at the time of marriage and if it is neglected may lead to serious consequences. It is unfortunate that leucorrhea of married women is so often regarded only as a necessary inconvenience. Its possible character and the probable outcome if disregarded should be impressed upon every woman.

The danger to the young wife is so great that special examination of the prospective husband who has once had gonorrhea should always be insisted upon before marriage is consented to.

Vaginal discharge in women past the menopause is often disregarded because of the popular belief that women at this period of life are exempt from disease of the generative apparatus.

The most frequent causes of vaginal discharge at this period are senile endometritis and cancer of the uterus. Both of these conditions are important; senile endometritis because it leads to constant ill health or chronic invalidism and premature aging; and cancer because it endangers the life of the patient. *Hence at no period of a woman's life is investigation of a vaginal discharge more important than after the menopause.*

It is unfortunate that the term leucorrhea is so indiscriminately used to designate any and all forms of discharge from the female genitalia. It is so frequently given as a symptom by the patient who consults the physician that it has come to be regarded as natural, and its actual character is seldom noted.

